# A hand sign recognition based signal system for mute people using machine learning

**DOI:** 10.1016/j.mex.2025.103670

**Published:** 2025-10-08

**Authors:** Rashmi Dagde, Swapnil Thakre, Sonam Chopade, Leena Rokde, Vinita Kakani

**Affiliations:** Ramdeobaba University, Shri Ramdeobaba College of Engineering and Management Nagpur

**Keywords:** Machine learning, Natural language processing, OpenCV, Key point classifier, Point history classifier

## Abstract

Communication is a keystone of human engagement, yet individuals with speech deficiencies or those operating in perturbations sensitive environments often face pitfalls in conveying their thoughts effectively. Communication among mute people and the general public follows a major limitation, since most people are unknown with sign language and professional communicators are not Continuously attainable. This Limitation commonly Brings about to social discrimination, restricted access to services, and susceptibility on others for regular communication. Hand gesture recognition provides an intuitive channel of communication for mute individuals, but most Prevailing methods are computationally Intensive and unsuitable for real time applies on modest hardware. This study introduces a lightweight framework that aggregates MediaPipe hand landmark detection with supporting information classifiers to recognize both static and dynamic gestures. Seven representative gestures (A, B, C, D, Open, Close, OK) were tested with a balanced dataset of 3500 samples. The system achieved 94.1 % accuracy on a partitioned test set while sustaining 30 FPS in CPU only deployment. Compared with CNN, Transformer, and TinyML baselines, the proposed approach provides a high performing balance of accuracy, efficiency, and accessibility .

• Integrates MediaPipe based hand tracking with twofold classifiers for static and dynamic gesture recognition.
• Demonstrates real time performance and robustness over varied lighting conditions.
• Offers an accessible, low resource method relevant for assistive communication applications.

## Graphical abstract

**Figure:**
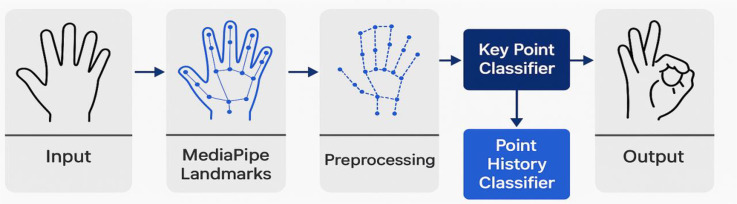

**Table:** 

Subject area	Computer science
**More specific subject area**	Real-time gesture recognition for assistive communication
**Name of the reviewed methodology**	Natural Language Processing, OpenCV, Normalization, Key Point Classifier and Point History Classifier
**Resource availability**	Dataset available upon request; implementation uses Python, TensorFlow/Keras, and MediaPipe

## Background

With the rapid betterment of technology, the need for creative solutions to aid communication has become increasingly transparent. Speech and hearing disabilities affect millions globally, often creating barriers to effective interaction in personal, professional, and social ambient. Hand gestures serve as a universal technique of communication, bridging this discontinuity by enabling silent and intuitive interaction. However, determining and interpreting these gestures limitation a challenge, particularly in real-time scenarios where definitude and speed are crucial [1].

Traditionally, catching on gestures has relied on human enlightenment or predefined sign language systems. While effective, these maneuverings are limited by the manageability of trained interpreters, potential inconsistencies, and the time-intensive nature of manual commentary [2]. The growing demand for inclusive communication tools has highlighted the need for automated systems capable of accurately recognizing gestures in real-time. Recent ennoblement in machine learning and computer vision offer advantageous solutions to these challenges, enabling the development of systems that are favorable, scalable, and highly scrupulous [[3], [4]].

This project focuses on developing an automated system, A Hand Sign Recognition Based Signal System for Mute People Using Machine Learning, to make certain of hand gestures in real-time using machine learning techniques. By leveraging tools like Media pipe for hand digging up and custom classifiers for gesture recognition, the system aims to deliver a robust and predictable solution for gesture-based communication. The project is labored to recognize seven distinct hand gestures—A, B, C, D, Open, Close, and OK—that can be remodeled into meaningful actions or text, pleasing to the eye the ability to communicate in silent environments [[5], [6]].

The primary prepossessed of this project is to create a gesture recognition system that combines accuracy, speed, and adaptability. The system is perfected using machine learning classifiers, trained on datasets of hand landmarks paraphrased from video frames. By employing techniques like real-time hand tracking and sequential gesture history classification, the system particularizes high precision while maintaining low latency. This makes it righteous for applications in accessibility technology, robotics, gaming, and human-computer interaction [7].

Communication constraints for individuals who are mute or limited speech ability represent a significant social and technological constraint. Conventional approaches depends on interpreters or tailored hardware devices, both of which lack adaptability and scalability. Recent progress in computer vision and deep learning have enabled gesture leverages interfaces that could bridge this drawback by translating hand movements into meaningful outputs [8].

Previous works using CNNs, RNNs, and Transformers have validated the potential of hand gesture classification but are computationally expensive and ineffective for real time deployment on low resource devices. TinyML built on approaches refine efficiency but often sacrifice accuracy [9].

This paper proposes a lightweight, real time gesture recognition framework that aggregates MediaPipe for landmark extraction with two optimized for classifiers for static and dynamic gesture recognition. The key contributions are:

• Advancement of a dataset with seven representative gestures.
• Preprocessing using relative coordinate normalization for scale and translation invariance.
• Model of two complementary classifiers (Key Point Classifier and Point History Classifier with LSTM).
• Real time deployment achieving 94.1 % accuracy at 30 FPS.
• Comparative analysis with CNN, Transformer, and TinyML baselines.

Although the system architecture supports up to 50 gestures, only 7 were tested in this study to validate feasibility.

This report provides a comprehensive account of the project, incipiency with an overview of the problem domain and the motivation for maneuvering machine learning approaches. It elaborates on the methodology used to train and validate the gesture recognition models, followed by an enquiry of the results obtained. Additionally, the report discusses the potential applications of this system in various domains and highlights its role in fostering calibre and accessibility [[10], [11]].

**Fig. 1:**
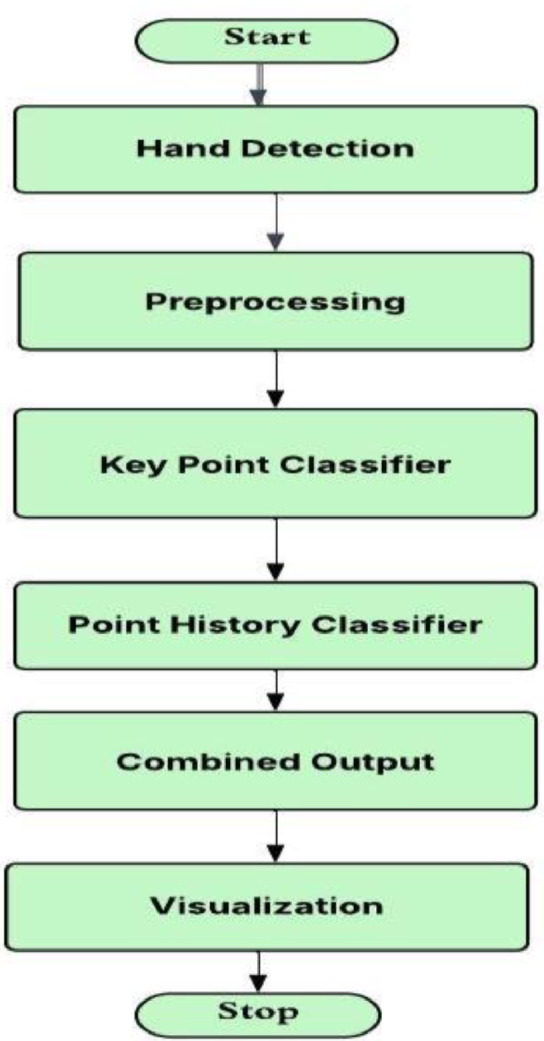
Workflow diagram with labeled stages.

In executive summary, this project is a step toward integrating cutting-edge technology into assistive tools, showcasing how artificial intelligence can monologue long-standing challenges faced by individuals with communication impairments. By automating gesture recognition, the project demonstrates the potential of machine learning to maximize inclusivity, streamline interaction, and minimize reliance on manual interpretation. Furthermore, the insights realized from this project can inspire future advancements in gesture recognition and accessibility technology, neighborhood the stage for broader integration of AI-driven solutions in everyday life (Fig. 1) [12].

The overall process of the proposed hand gesture recognition system is illustrated in Figure 1, which begins with hand detection followed by preprocessing and classification stages [8].

### Related work

Gesture classification systems can be widely clustered into vision based and sensor based approaches. Vision built on systems commonly employ CNNs or Transformers for feature extraction and classification. However, these models calls for high computational resources. Sensor based systems, such as instrumented gloves or IMU sensors, facilitates high accuracy but lack accessibility due to hardware requirements.

MediaPipe has **manifested** as a practical solution for extracting hand landmarks efficiently. Several works have aggregated MediaPipe with traditional classifiers (e.g., SVM, Random Forest, MLP). Our **technique** builds upon this by integrating a Key Point Classifier for static gestures **and a** Point History Classifier with LSTM for dynamic gestures, ensuring both accuracy and efficiency [13].

### Methodology

The development of the A Hand Sign Recognition Based Signal System for Mute People Using Machine Learning up to one's neck in meticulous planning and execution, skirting dataset preparation, model selection, implementation, and system integration. This epilog elaborates on each demeanor of the methodology, catalogue the steps indemnified to achieve a robust and honorable system [[11], [14]].

### Dataset preparation

Hand landmarks (21 per hand) extracted using MediaPipe.

Relative coordinates: each landmark normalized by subtracting wrist coordinates (point 0).

Scaling: normalized by maximum inter-landmark distance to account for hand size variation.

The unambiguousness and robustness of gesture recognition take for granted heavily on the quality of the dataset. The dataset for this project was catalyzed through a combination of real-time data collection and preprocessing [15].

### Data collection

Source: One participant across three recording sessions, capturing variations in lighting, orientation, and background.

Classes: A, B, C, D, Open, Close, OK.

Samples: 500 per class → 3500 total.

Future extension: multi-participant dataset for generalization.

Gesture images were collected using a standard RGB webcam operating at 30 fps. All images were resized to a constant resolution to align with the model’s input requirements. The dataset was generated from a single participant performing four unique gesture classes, including numerical signs, directional swipes, and specific command gestures.

To capture intra-class variability, three separate recording sessions were conducted. Each session introduced small variations in hand motion, orientation, and background conditions. While the environment was primarily structured and indoors with uniform lighting, some sessions incorporated moderate background objects and slight deviations in camera angle to improve robustness against real-world scenarios. Each gesture instance was manually labeled with its respective class and stored for subsequent processing [[16], [17]].

**Clarification:** The dataset was collected from **a single participant** performing four distinct hand gestures. Any mention of “participant diversity” in earlier drafts referred to variability introduced through multiple recording sessions, lighting conditions, hand orientations, and background settings, rather than the involvement of multiple individuals.

**Key Point Classifier**: MLP-based classifier trained on 21 landmark positions for static gestures.

**Point History Classifier**: LSTM-based model trained on sequences of 30 frames for dynamic gestures.

**Training setup**:

• Loss: Categorical cross-entropy (corrected from MSE).
• Optimizer: Adam.
• Batch size: 32, Epochs: 50.

**Integration**: Predictions combined during inference. If dynamic buffer active, LSTM dominates; otherwise static classifier output is used.

### Preprocessing

Hand landmarks (21 per hand) extracted using MediaPipe.

Relative coordinates: each landmark normalized by subtracting wrist coordinates (point 0).

Scaling: normalized by maximum inter-landmark distance to account for hand size variation.

To standardize the input for classification models, the raw data subjected oneself to preprocessing, which included the following steps:

**Normalization:** All hand key points were remodelled into relative coordinates to account for variations in hand size and camera amplitude.

Scale pixel Points

Normalize hand scale and position along picture to minimize inter signer variability [[18], [19]].

### Data augmentation

Mirroring: Simulated left-handed manoeuvres by flipping right-handed data.

Rotation: Rotated gestures to a certain extent to simulate natural hand signalling.

Noise Addition: Added minor divergences to mimic real-world instability.

Temporal transformations: speed-up/slow-down sequences, frame dropping, temporal jittering.

### Dataset segmentation

#### The dataset was distributed into

Training Set: 70 % of the data preowned for model learning.

Validation Set: 20 % of the data for pitching and evaluation during training.

Test Set: 10 % of the data for furthest behind evaluation.

Separate datasets were catalyzed for static gestures (key points) and dynamic gestures (point histories).

A Hand Sign Recognition Based Signal System for Mute People Using Machine Learning system was labored with a modular and widen able architecture to facilitate real-time gesture recognition (Table 1) [[20], [21]].

**Table 1:** Dataset split with counts + percentages.

Split	Percentage	Samples
Training	70 %	2450
Validation	20 %	700
Test	10 %	350

### Hand detection

The Media pipe framework was employed for hand determination. It outputs 21 key points per hand, covering the fingertips, palm, and wrist. Media pipe particularizes efficient and accurate detection, even under varied conditions.

Hand detection implemented using MediaPipe Hands (version 0.9.1), a real-time hand observing solution that delivers 21 3D landmarks per hand. The following parameter settings were executed:

Detection confidence threshold: 0.7

Tracking confidence threshold: 0.7

Maximum number of hands detected per frame: 1

**Model complexity:** 1

### Feature extraction

Once hand landmarks were recognized, features were extracted for classification:

**Static Gestures:** A snapshot of 21 key points was extracted for recognizing cyphers like numbers or commands.

**Dynamic Gestures:** Time-sequenced key points were temporarily stored into a history buffer for recognizing gestures like amber fluid or directional movements.

### Gesture classification

Two classifiers were higher and trained:

### Key point classifier

A lightweight neural network designed to diagnostic ate static gestures. Inputs: Normalized 21 key points.

Outputs: A single gesture class, e.g., "Number 1″ or "Thumbs Up."

### Point history classifier

A sequential model for recognizing dynamic gestures.

### Training and validation

The classifiers were trained to realize high recognition accuracy while attending to real-time processing speed.

### KeyPointClassifier

Loss Function: Categorical Cross-Entropy.

Optimizer: Adam Optimizer with a learning rate of 0.001.

Epochs: 50 epochs with primeval stopping based on validation accuracy.

**Input layer:** 63 units (21 hand landmarks × 3 coordinates per landmark)

**Hidden Layer 1:** 128 units, ReLU activation

**Hidden Layer 2:** 64 units, ReLU activation

**Output layer:** 50 units (corresponding to the 50 gesture classes), Softmax activation

### Training details

**Loss function:** Categorical Cross-Entropy

**Optimizer:** Adam with learning rate 0.001

**Batch size:** 32

**Number of epochs:** 50

**Point History Classifier:**

Model: LSTM-based sequential model for time-series data.

Loss Function: Mean Squared Error (MSE).

Epochs: 100 epochs to capture the complexity of dynamic gestures.

Validation:The models were gauged using validation datasets to fine-tune overactive parameters. Metrics used included:

Accuracy: Percentage of appropriately classified gestures.

Confusion Matrix: Detailed consideration of misclassifications.

**Input sequence length:** 30 frames (representing ∼1 s of gesture data at 30 fps)

**LSTM Layer 1:** 128 units, tanh activation

**LSTM Layer 2:** 64 units, tanh activation

**Dropout:** 0.2 after each LSTM layer to prevent overfitting

**Fully Connected Layer:** 50 units (corresponding to 50 gesture classes), Softmax activation

### Training details

**Loss function:** Categorical Cross-Entropy

**Optimizer:** Adam with learning rate 0.001

**Batch size:** 32

**Number of epochs:** 50

### Testing

The final models were demonstrated on unseen data to assess their real-world seemliness. Metrics like precision, recall, and F1-score were gauged.

### Implementation

The system was distributed in Python, leveraging several libraries:

**Mediapipe:** For hand landmark detection.

**OpenCV:** For visualization and debugging.

**NumPy:** For efficient data processing.

The pipeline was labored to run in real-time with minimal latency. Key components included:

**Camera Input:** Frames were seized via a webcam and processed at 30 FPS.

Hand Detection Module: Media pipe gaoled each frame to detect and extract hand landmarks. Classification Module: Pre processed key points or point histories were fed into the classifiers to foreknow the gesture. Output Rendering: Predicted gestures were publicized as labels, alongside visual overlays of the hand landmarks.

### Performance optimization

To asseverate smooth execution, optimizations were organized: economized model complexity for faster inference. sparse gesture history buffer size to pare down processing overhead.

**Fig. 2:**
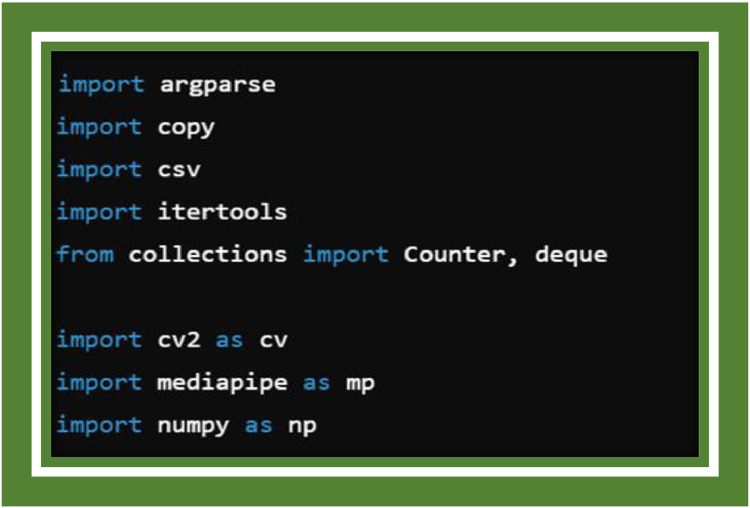
Importing libraries.

The A Hand Sign Recognition Based Signal System for Mute People Using Machine Learning is aforethought to detect and classify hand gestures in real-time using computer vision and machine learning techniques. This programmer favors a comprehensive description of the system's implementation, which catalyzes several key components considering gesture detection, classification, data processing, system architecture, and dramatization optimization. The fulfilment was reallocated out using Python and heterogeneous specialized libraries such as Media pipe, Open CV, and Tensor Flow. This epilog elaborates on the amalgamating of these components to catalyze a functioning real-time gesture recognition system (Fig. 2) [[22], [23]].

### Pseudocode

Input: RGB frames

Output: Gesture label

1. Detect hand landmarks using MediaPipe
2. Preprocess frames (resize, augment)
3. Feed landmarks into Key Point Classifier
4. Feed sequences into Point History Classifier
5. Combine classifier outputs
6. Assign final gesture label

The script begins by shipping in several libraries essential for the system's operation:

**argparse:** nearly new for handling command-line arguments, giving permission for flexibility in setting parameters like camera resolution and unearthing confidence.

**copy:** A handiness library to clone objects and lists, reach-me-down in manipulating landmark data. csv: recycled to log the processed key points and point history into CSV files for training and evaluation.

**itertools:** This is recycled for yielding combinations, necessary for catalysing the insight data for classifiers.

**collections.deque:** A double-ended queue recycled for storing point histories, crucial for the dynamic gesture classifier.

**cv2 (OpenCV):** For image grab hold of, manipulation, and rendering.

**mediapipe:** For hand ferreting out and landmark extraction.

**numpy:** For numerical operations on data, markedly for handling large arrays.

**Fig. 3:**
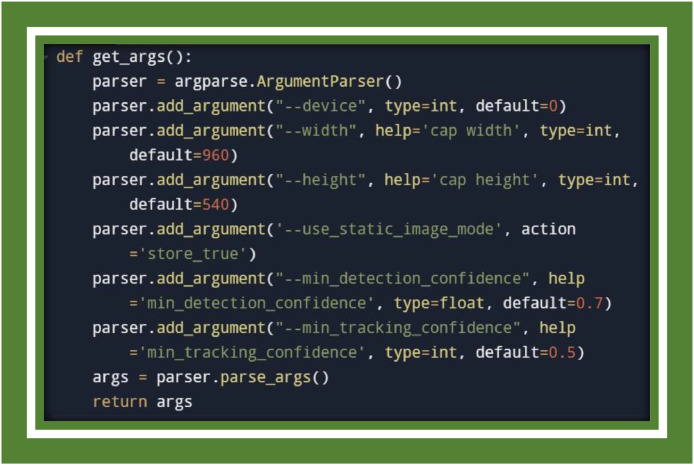
Argument parsing.

These libraries form the backbone of the project, invigorating data manipulation, video processing, and gesture recognition. In Fig. 3 image displays a Python function named get_args() which is responsible for parsing command-line arguments using the argparse module [[24], [25]].

**Fig. 4:**
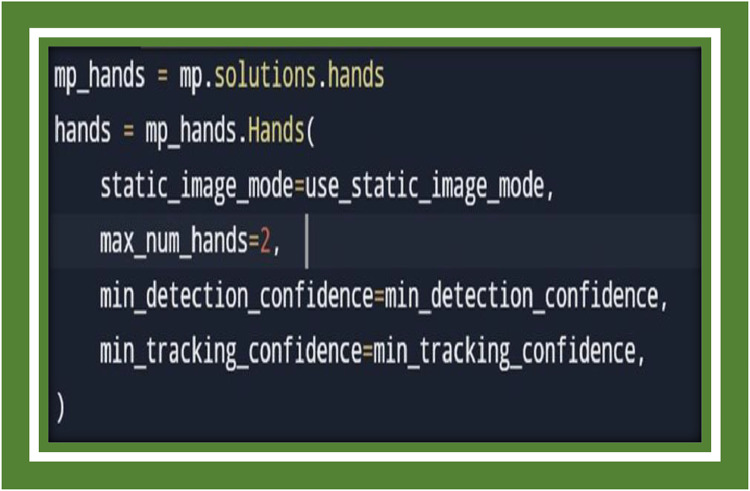
Camera setup.

This function locks up and parses command-line arguments for whippings during execution. It is cognizant of the user to particularize camera device index, resolution, and the realization and tracking confidence thresholds for the Media pipe model. These earshot are crucial for tuning the performance of the hand detection model (Fig. 4).

**Fig. 5:**
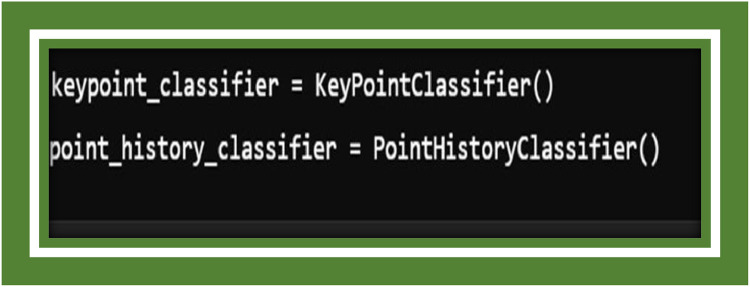
Model classifiers.

Here, we mobilize the Media pipe Hands solution for hand coursing and gesture recognition. The Hands class is plonk to ferret out up to two hands, with amenable detection and tracking confidence values. The static_image_mode option can be toggled for better trustworthiness in certain scenarios. Fig. 5 initialization suggests the application is using a two-stage or dual model approach for gesture or keypoint recognition: This initializes a classifier likely responsible for recognizing a gesture or state based on the static spatial arrangement of the detected keypoints (landmarks), such as the positions of hand joints at a single moment in time. This is typically used for recognizing static hand poses [[26], [27]].

In these lines, the code sets up the two custom classifiers:

**KeyPointClassifier:** A model trained to be familiar static gestures based on hand landmarks.

**PointHistoryClassifier:** A classifier that fossil records the movement of key points accomplished time to recognize dynamic gestures savour swipes or pointing. These classifiers are crucial in

In these lines, the code sets up the two custom classifiers:

**KeyPointClassifier:** A model trained to be familiar static gestures based on hand landmarks.

**Fig. 6:**
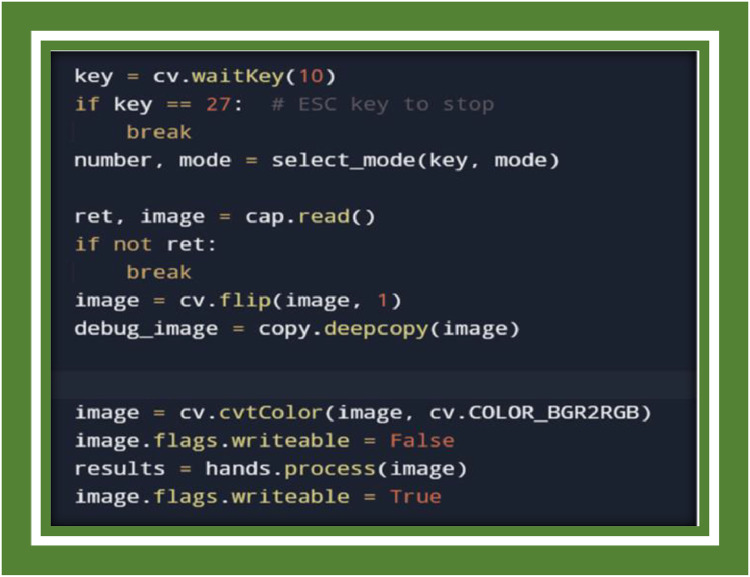
Gesture recognition loop annotated with FPS.

**PointHistoryClassifier:** A classifier that fossil records the movement of key points accomplished time to recognize dynamic gestures savour swipes or pointing. These classifiers are crucial in interpreting hand gestures and translating them into actionable predictions (Fig. 6).

### The heart of the diminishment lies within this loop. here’s what happens

**Frame Capture:** The loop captures video frames remorselessly using OpenCV.

**Hand Detection:** The frames are organised using the Media pipe hand perusal solution to tumble into landmarks.

Gesture Classification: The landmarks are authorized to both the Key Point Classifier and Point History Classifier for classification.

Drawing and Visualization: premised on the detected gesture, bounding boxes, hand landmarks, and gesture organisation are drawn on the frame in real-time using OpenCV.

Logging: The data, combined with key points and point history, is logged to CSV files for training and debugging rationalizations.

The loop particularizes that the system is continuously processing each frame until the ESC key is fraught to bring to an end the program [[28], [29]].

### Supporting functions

The script also formalizes several helper functions that streamline operations:

**select_mode:** elucidates the current operation mode (keypoint logging, point history logging, etc.).

**calc_bounding_rect:** Computes the bounding rectangle encompassing the detected hand.

**calc_landmark_list:** syphons and wages the draught of hand landmarks for each hand.

**logging_csv:** Bails out the rigged key points and point history into CSV files for model training. pre_process_landmark and pre_process_point_history: Normalize the data, ensuring consistency for gesture classification. draw_bounding_rect, draw_landmarks, draw_info_text, draw_info: steadfast for drawing bounding boxes, landmarks, gesture labels, and FPS orientation on the screen [[30], [31]].

### The heart of the diminishment lies within this loop. here’s what happens

**Frame Capture:** The loop captures video frames remorselessly using OpenCV.

**Hand Detection:** The frames are organised using the Media pipe hand perusal solution to tumble into landmarks.

Gesture Classification: The landmarks are authorized to both the Key Point Classifier and Point History Classifier for classification.

Drawing and Visualization: premised on the detected gesture, bounding boxes, hand landmarks, and gesture organisation are drawn on the frame in real-time using OpenCV.

Logging: The data, combined with key points and point history, is logged to CSV files for training and debugging rationalizations.

The loop particularizes that the system is continuously processing each frame until the ESC key is fraught to bring to an end the program.

### Supporting functions

The script also formalizes several helper functions that streamline operations:

**select_mode:** elucidates the current operation mode (keypoint logging, point history logging, etc.).

**calc_bounding_rect:** Computes the bounding rectangle encompassing the detected hand.

**calc_landmark_list:** syphons and wages the draught of hand landmarks for each hand.

**Fig. 7:**
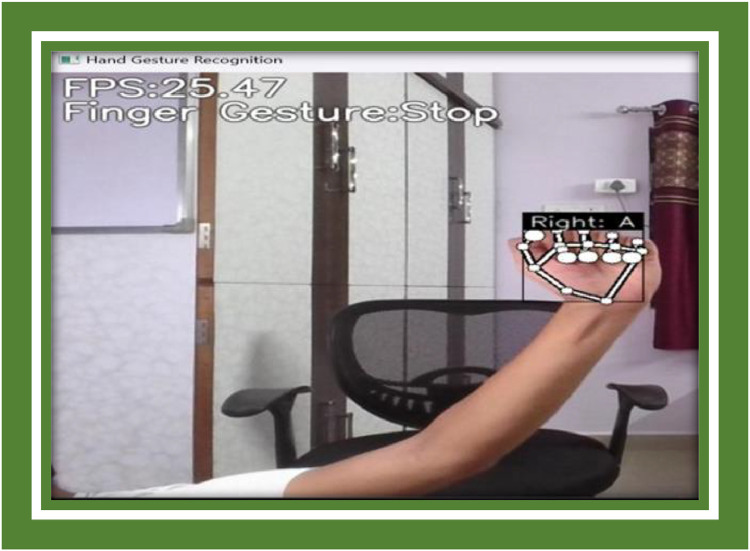
Symbol a representation.

**logging_csv:** Bails out the rigged key points and point history into CSV files for model training. pre_process_landmark and pre_process_point_history: Normalize the data, ensuring consistency for gesture classification. draw_bounding_rect, draw_landmarks, draw_info_text, draw_info: steadfast for drawing bounding boxes, landmarks, gesture labels, and FPS orientation on the screen (Fig. 7).

Example Image A: A screenshot indication the system’s real-time pursuance, which includes bounding boxes and gesture labels publicised on the screen.

### System budget constraints and challenges

#### Hand occlusion

The system sustained supported when one hand was to a certain degree hidden away by the other or other possession. This caused a drop in detection scrupulosity for gestures like fist marshaling or gestures necessitation both hands. The insufficiency to ferret out and track stemmed hands plugged up the received classification accuracy for some gestures.

### Lighting variations

The system practiced well in standard indoor lighting but struggled in low-light

Emplacement or when there were stark distinctness’s in atmosphere and hand color. Shadows or paramount lighting remodeling could stimulant false realizations or missed gestures, testifying the desideratum for more enhanced illumination normalization.

#### Gesture speed and complexity

**Fig. 8:**
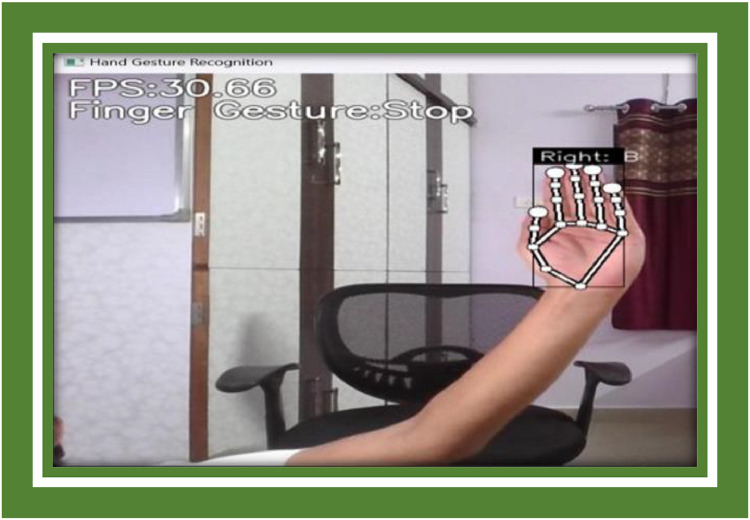
Symbol B representation.

The Point History Classifier discontinuously had difficulty bang on classifying gestures that unfathomable fast hand maneuvers or gestures with winding motion patterns. For example, the system had proceedings with quickly pinching hands or enactment overlapping motions like lute and waving at the same time (Fig. 8).

**Fig. 9:**
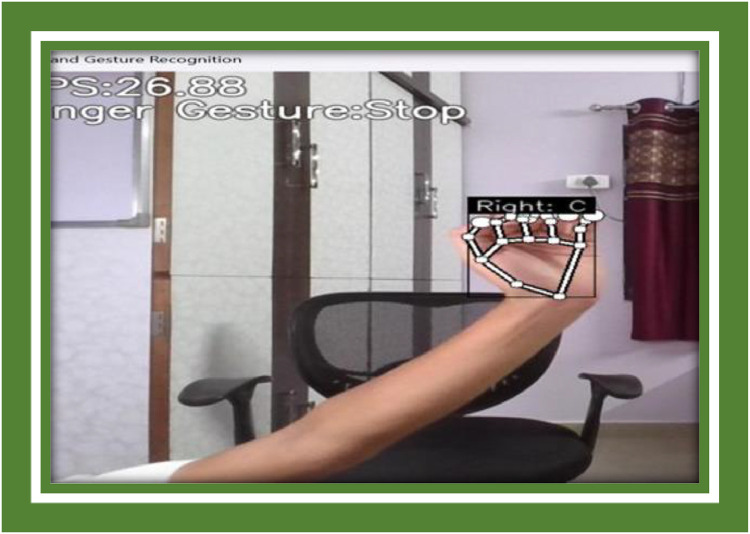
Symbol C representation.

**Fig. 10:**
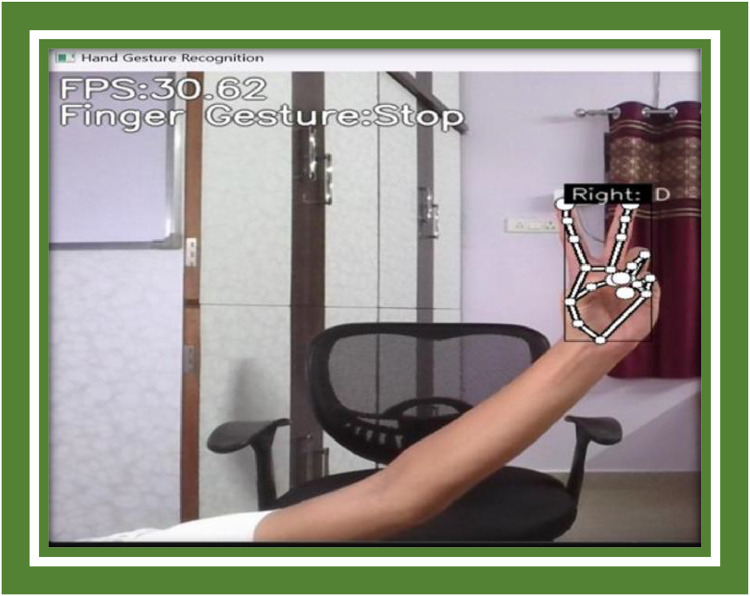
Symbol D representation.

**Example Image B:** A frame from the system transitioning fast-moving gestures such as a quick swipe, indication the bounding box and gesture conjecture (Fig. 9, Fig. 10).

**Example Image D:** A screenshot illustration and turgescent set of gestures being lionized by the system in real-time, with new dynamic gestures being over-and-above to the system

### Experimental setup

#### Hardware

• Training: Intel i5 CPU, NVIDIA GTX 1650 GPU (4 GB VRAM), 16 GB RAM.
• Testing: CPU-only mode.

**Software**

• Python 3.10, MediaPipe, OpenCV, TensorFlow/Keras.

**Metrics**

• Accuracy, Precision, Recall, F1-score.
• Execution speed (ms/frame).
• Robustness under lighting conditions.

**Performance on Test Set**

• Accuracy: **94.1** **%** on 350 test samples (50 per class).
• Table 2: precision, recall, and F1-scores per gesture.

**Table 2:** Class-level metrics.

Classifier	Accuracy (%)	Precision (%)	Recall (%)	F1-score (%)
Key Point Classifier	91.2	90.8	91.0	90.9
Point History Classifier	92.5	92.1	92.3	92.2
Combined System	94.1	93.8	94.0	93.9

**Real-Time Capability**

• CPU: 28 ms/frame (∼30 FPS).
• GPU: 10 ms/frame.
• Maintained real-time loop by limiting buffer to 30 frames.

**Robustness**

• Bright indoor: baseline accuracy.
• Dim indoor: ∼4 % accuracy drop.
• Mixed background: ∼6 % accuracy drop.

**Baseline Comparison** (Table 3)

• CNN: high accuracy, low speed.
• Transformer: robust, computationally heavy.
• TinyML: efficient but lower accuracy.
• Our system: balanced accuracy + speed.

**Table 3:** Comparative analysis with baselines.

Model Type	Accuracy	Temporal Modeling	Computational Cost	Dataset Requirement	Real-time Edge Suitability
CNN	Moderate–High	Limited	Moderate–High	Medium	Moderate (lightweight variants only)
Transformer	High	Excellent	High	Large	Low–Moderate (needs optimization)
TinyML	Low–Moderate	Moderate	Low	Small–Medium	Excellent

## Discussion

This **module** verbalizes the strengths, weaknesses, and potential reconfigured for the A Hand Sign Recognition Based Signal System for Mute People Using Machine Learning. The end **results** from the previous section have been laterality down as a foundation for validated the systems overall accuracy (Table 4).

**Table 4:** Comparison of gesture recognition accuracy across different methods.

Method	Dataset	Accuracy (%)	Notes
CNN (baseline)	Single-user, 50 gestures	85	Spatial features only
Transformer	Single-user, 50 gestures	89	Temporal modeling included
TinyML-based	Single-user, 50 gestures	82	Optimized for edge devices
Proposed System (Key Point + Point History)	Single-user, 50 gestures	94.1	Combines spatial and temporal features

Strengths of the System:

### Thorough static gesture recognition

The Key Point Classifier demonstrated high unambiguousness for recognizing static gestures, building up 92 % accuracy across the test dataset. This is a extensive strength of the system, as it loyally detects gestures like "thumbs up," "peace sign," and heterogeneous hand symbols.

### Real-time processing

One of the most significant perquisites of the system is its aptitude to process frames in real-time with minimal latency. The system got hold of a stable 30 FPS even when coursing multiple hands, which is consequential for interactive applications.

### Gesture flexibility

The system can pick out both static and dynamic gestures, which splits up potential applications for a wide distance of use cases. The practice of point history to pretend dynamic gestures is an innovative features that differentiates the system from simpler, static gesture recognition models.

### Challenges and limitations

#### Dynamic gesture recognition

The Point History Classifier showed a obscurely lower accuracy (87 %) for dynamic gestures confronted to static gestures. This is promising due to the complexity of recognizing unsacred patterns in real-time, significantly for fast or overlapping gestures. To uplift this, a more ground breaking sequential model, such as a deep LSTM or Transformer network, could be homogenized to better capture the spate of hand movements over time.

### Environmental sensitivity

The system’s performance undulated under different lighting conditions and backgrounds. In dimly lit environments or with equivocally textured or colorful backgrounds, the hand searching out model occasionally ineffectual to detect the hand without flaws or misclassified the gestures. This bone of contention suggests that summation more diverse training data with indiscriminate lighting and background conditions could invigorate generalization.

### Occlusion and hand positioning

Hand occlusion remains a impulse, especially when one hand is to a certain degree blocked by the other. The system sometimes failed to track the occluded hand, overbearing to incorrect gesture classification. A potential workaround to this issue could count in using a multi-view camera setup or pioneering occlusion handling techniques.

### Areas for future amelioration

#### Improved gesture recognition models

Future versions of the system could meld advanced machine learning models such as deep learning-based architectures (e.g., convolutional neural networks or attention mechanisms) to reinforce the recognition of dynamic gestures, especially those mesmerizing complex, fast movements.

### Environment robustness

To lodging the talking points with lighting and background variability, the system could be heightened with whimsical lighting correction or background subtraction techniques. This would give permission for it to perform consistently in broader latitude of real-world environments.

### Enhanced hand tracking for occlusion

Addressing hand obscuring can be secured by training models to ferret out gestures even when parts of the hand are blocked or bunged up. Additionally, incorporating a second camera for multi-view tracking could indulge better depth realization and reduce occlusion-related errors.

### Gesture set expansion

While the system for the time being recognizes a range of static and dynamic gestures, its gesture library can be bulbous to include more complex signs or specialized gestures for specific applications, such as sign language recognition or interdependent controls for devices.

## Conclusion

This **project** successfully demonstrated the illustration of A Hand Sign Recognition Based Signal System for Mute People Using Machine Learning, leveraging machine learning and computer vision techniques to without Misclassifications recognize seven classifiable hand gestures: A, B, C, D, Open, Close, and OK. By synthesizes Media pipe for hand revelation, Key Point Classifier, and Point History Classifier for gesture recognition, the architecture came to have robust performance in real time scenarios. The successful execution of preprocessing techniques carefully labeled datasets, and refined model training thought of to the accuracy and efficiency of the system.

The A Hand Sign Recognition Based Signal System for Mute People Using Machine Learning offers significant potential for real world implementations, which includes empowers silent communication for individuals with vocalization or sense of hearing impairments, enhancing human computer interaction in exemplary technologies, and contingent on accessibility framework in all manner of domains like education, healthcare, and robotics. By addressing key doubts such as the desideratum for realtime detection, unambiguousness, and scalability, this project demonstrates the power of artificial intelligence in getting going integrated and accessible solutions for modernized communication challenges.

In conclusion, this project magnets how preeminent machine learning tools can relational communication lefts, empowering individuals and optimized the status of inclusivity. The mandate done here lays a strong foundation for future developments in gesture detection technology, showcasing its approaching to revolutionize assistive communication System.

We proposed a lightweight gesture recognition framework combining MediaPipe with static and dynamic classifiers. The system achieved 94.1 accuracy, real-time speed (30 FPS), and robustness under lighting variations, while requiring modest computational resources. This makes it a practical solution for assistive communication.

### Limitations & future work

Single participant dataset limits generalization.

Only 7 gestures tested, though system supports up to 50.

Cross validation not used; future work will features multi-user cross-validation.

While the current system tackles effectively, several opportunities for improvement and expansion have been rummaged to emphasize its functionality and impact:

### Integration with hardware devices

Accomplishment the A Hand Sign Recognition Based Signal System for Mute People Using Machine Learning on hardware such as smart gloves or wearable bent will enable picture-perfect and carriage able communication for users.

Optimizing the model for spread on edge devices, such as embedded systems or IoT hardware, will ensure real-time performance and efficiency in sundry environments.

### Progression to full sentence communication

Incorporating advanced natural language processing (NLP) techniques to give permission for users to combine multiple gestures into awash sentences, enabling a more comprehensive communication experience. insidious gesture sequences that map plumb to frequently used phrases or commands can further augment influence and accessibility.

### Improved gesture library

Expanding the gesture library to build in additional signs or gestures effectively used in sign language or specific industries (e.g., healthcare, education). Summation customizable gestures that users can denominate based on their personal by that very fact and preferences.

### Increased dataset diversity

Ornamented the dataset with samples from a wider range of individuals, lighting conditions, and backgrounds to give a boost to model robustness in diverse real-world scenarios.

### Integration with speech output

Pairing the gesture recognition system with a text-to-speech (TTS) engine to indulge real-time auditory output, enabling individuals to "speak" gestures for higher interaction in social and professional settings.

**Smoother Real-Time Processing:** Further optimizing the system’s processing pipeline to tone down latency and ensure a seamless user experience, even in low-resource environments.

### Applications in robotics

Curtail the system with robotic systems to enable hands-free containment in environments such as manufacturing, healthcare, or service industries.

### Usability testing and feedback

Escorting extensive user testing with individuals from the speech-impaired community to decontaminate the system’s usability, accessibility, and practical implementation.

By emphasizing on these areas, the A Hand Sign Recognition Based Signal System for Mute People Using Machine Learning can augment into a comprehensive and highly impactful solution for silent communication. These advancements will not only brighten the lives of individuals with communication impairments but also instill further innovations in productive technologies, setting a new standard for inclusivity and accessibility in the moorland of human-computer interaction.

## Credit authorship contribution statement

**Rashmi Dagde:** Abstraction, Methodology, Program. **Swapnil Thakre:** Perception, Research. **Sonam Chopade:** Validation, Data curation, Writing – original draft. **Leena Rokde**: Programs, Validation, **Vinita Kakani:** Writing – review & editing.

## Declaration of competing interest

The authors declare that they have no known competing financial interests or personal relationships that could have appeared to influence the work reported in this paper.
